# Effects of C-Terminal Carboxylation on α-Conotoxin LsIA Interactions with Human α7 Nicotinic Acetylcholine Receptor: Molecular Simulation Studies

**DOI:** 10.3390/md17040206

**Published:** 2019-04-02

**Authors:** Jierong Wen, Andrew Hung

**Affiliations:** School of Science, College of Science, Engineering and Health, RMIT University, Melbourne, VIC 3001, Australia

**Keywords:** homology modeling, MD simulation, α-conotoxin, nicotinic acetylcholine receptor, C-terminal amidation/carboxylation

## Abstract

α-Conotoxins selectively bind to nicotinic acetylcholine receptors (nAChRs), which are therapeutic targets due to their important role in signaling transmission in excitable cells. A previous experimental study has demonstrated that carboxylation of the C-terminal of α-conotoxin LsIA reduces its potency to inhibit human α7 nAChR relative to naturally amidated LsIA. However, little is known about the contribution of conformational changes in the receptor and interactions, induced by C-terminal amidation/carboxylation of conotoxins, to selective binding to nAChRs, since most conotoxins and some disulfide-rich peptides from other conotoxin subfamilies possess a naturally amidated C-terminal. In this study, we employ homology modeling and molecular dynamics (MD) simulations to propose the determinants for differential interactions between amidated and carboxylated LsIAs with α7 nAChR. Our findings indicate an overall increased number of contacts favored by binding of amidated LsIA versus its carboxylated counterpart. Toxin-receptor pairwise interactions, which may play a role in enhancing the potency of the former, include ARG10-TRP77, LEU141 and CYS17-GLN79 via persistent hydrogen bonds and cation-π interactions, which are weakened in the carboxylated form due to a strong intramolecular salt-bridge formed by ARG10 and carboxylated C-terminus. The binding of amidated LsIA also induces enhanced movements in loop C and the juxtamembrane Cys-loop that are closely associated with receptor function. Additionally, the impacts of binding of LsIA on the overall structure and inter-subunit contacts were examined using inter-residue network analysis, suggesting a clockwise tilting of the α7 C and F loops upon binding to carboxylated LsIA, which is absent for amidated LsIA binding. The predicted molecular mechanism of LsIA binding to the α7 receptor may provide new insights into the important role of the C-terminal in the binding potency of conotoxins at neuronal nAChRs for pharmacological purposes.

## 1. Introduction

nAChRs are situated in the plasma membranes of certain neurons in the central and peripheral nervous systems [1]. These ligand-gated ion channels are involved in signal transmission, neuronal integration and cell excitability through activation by acetylcholine (ACh) binding [1]. There are two general types of nAChRs, heteropentamers and homopentamers, both forming a cylindrical helical bundle around the ion pore [2]. The homomeric nAChRs are constituted by 5 identical subunits, whereas heteropentameric nAChRs consist of distinct subunits which may include combinations of α1-10, β1-4, γ, δ and ε subunits [3]. The various combinations of subunits also render distinct nAChRs with unique physiological and pharmacological properties [4].

Human α7 nAChR subtype, as an example of homopentameric nAChRs, has been extensively studied due to its important function in cognitive processes and an implicated involvement in the cholinergic anti-inflammatory pathway [5]. The dysfunction of α7 nAChR, therefore, is related to many neurological diseases and disorders, such as schizophrenia [6,7], Alzheimer’s disease and Parkinson’s disease [1,8,9,10]. The determination of the crystal structures of acetylcholine binding protein (AChBP) complex to conotoxins has enabled the construction of molecular models of human α7 nAChR bound to conotoxins [6,11,12], although detailed structural information on toxin-bound nAChRs is currently relatively scarce. Even if the similarities between sequences of AChBP [13] and the extracellular domain (ECD) (the region for acetylcholine (ACh) binding [14]) of human α7 receptor are less than 30% [15,16], up to 52% of its sequence at the ligand binding domain (LBD) is identical to the ligand binding site of AChBP [17,18]. This suggests that AChBP and nAChRs share similar functional and structural properties [13,19], and that AChBP may serve as useful structural templates for comparative modeling of the ECDs of nAChRs.

Conotoxins, derived from marine cone snail, are peptides rich in disulfide bonds connecting conserved cysteine residues. The synthesis and manipulation of these neuroactive peptides as potential pharmacophores and molecular probes have attracted attention because of their relatively short length, well-defined backbone structure, and specificity to a wide range of ion channels and nAChRs with high potency [1,20,21,22]. Furthermore, with many possible variations of amino acid residues apart from conserved cysteines, conotoxins vary greatly in their ability to target different neuronal/muscular acetylcholine receptors [23]. The most studied conotoxin subfamily is α-conotoxins, that are known to naturally and selectively bind at nAChR subtypes that modulate the cholinergic pathway [24], as mentioned previously. α-Conotoxins commonly contain fewer than 20 amino acid residues with a compact structure including 2 disulfide bridges [25]. These peptides are widely utilized to probe the structure-function relationships of nAChR binding sites [26], for differentiating variable nAChR subtypes [5], and in the development of potential pharmacological tools [27].

**Figure 1:**
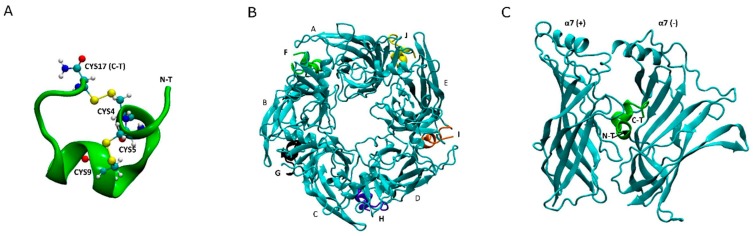
Ribbon conformation of amidated LsIA binding to human α7 nAChR. (**A**) The conformation of LsIA with four conserved cysteines. (**B**) Top view of LsIA binding at five interfaces between adjacent α7 subunits, where the subunits are shown in *cyan* color. (**C**) Side view of LsIA anchored to one interface of two adjacent α7 subunits.

α-conotoxin LsIA (17 amino acids in length), is a neuropeptide discovered from *Conus limpusi*, which shows distinct high binding affinity (IC_50_ = 10.1 nM) for targeting human (h) α7 nAChR subtype relative to α3β4 [28], α3β2 and α3α5β2 nAChR subtypes, as determined in previous experiments [29]. Similar to other α-conotoxins, LsIA possesses 2 disulfide bonds connecting 4 conserved cysteine residues [30], and the disulfide bridges produce a unique 2-loop structure (4/7) [23] with a naturally amidated C-terminal (shown in Figure 1A). The unique motif in loop 1 of certain α-conotoxins was implicated in subtype-selective binding to nAChRs via the highly conserved proline [31], such as Lo1a, GID and LsIA (in position 7 in LsIA) [1,29,32]. Other studies also pinpointed the importance of residues in the loop 2 on the specificity of α-conotoxins binding at nAChRs [33]. Moreover, it has been demonstrated that the C-terminus, ARG10 and connective ASNs (in position 12 and 13) of LsIA are involved in distinguishing between neuronal subtypes, namely, α3β4 [28], α3β2 and α7 nAChR [29], with mutations based on these residues showing distinct efficacies. It has also been shown that carboxylation of the C-terminus of α-conotoxin LsIA reduces potency to human α7 nAChR relative to naturally amidated LsIA, but enhances potency at α3β2 [29], highlighting the potential efficacy of C-terminus modification to alter nAChR subtype selectivity. Additionally, apart from most α-conotoxins, some peptides from other conotoxin subfamilies also retain a naturally amidated C-terminal, such as μ-conotoxin GIIIA [34] and KIIIA [35], ω-conotoxin, CVID [36] and MVIIA [37] that target ion channels and other proteins. Among them, MVIIA (ziconotide), is a strong analgesic for treating severe chronic pains, which has been approved by the US Food and Drug Administration (FDA) [38], meanwhile other conotoxins, such as ω-CVID [36] and α-Vc1.1 [39] for treatment of neuronal and cancer-related pains [40] are under clinical trials [41,42]. The possibility of altering the selectivity or potency via C-terminal carboxylation of these conotoxins, of known therapeutic importance, provides an additional exciting avenue for the development of novel conotoxin derivatives which may serve as molecular probes or new treatments.

Previous studies have mainly provided insights into determinants that affect the binding of some α-conotoxins to human α7 nAChR, in order to better understand the binding mechanisms of ligands [23,43], and structure-function relationships of α7 nAChR and α-conotoxins [1,44]. Additionally, much work has focused on mutating and synthesizing novel neuropeptides binding at the receptors with improved efficacy and specificity, experimentally or proposed via computational predictions [21,26,29,45].

It is widely accepted that α-conotoxins bind at the interface between two adjacent α7 subunits [18,46,47,48], where aromatic pockets are present for ACh targeting [49]. One example of binding conformation of α-conotoxin LsIA to human α7 nAChR is shown in Figure 1B,C. Compared with AChBP, highly conserved amino acids of α7 nAChR are contained within 7 loops, for which 3 loops (A–C) are located on the principal face, also termed the (+) face, and 4 loops (I–IV) on the complementary face, denoted as the (−) face [43], constituting the binding site of the ECD of α7 nAChR [50]. The key residues of these loops, therefore, play significant roles in interactions with key residues of α-conotoxins. The most important residue pairs that affect the stabilization of human α7 and α-ImI complex and human α7 and α-PnIB conformation are ImI-ARG7 and α7-TYR217(+), and PnIB-LEU10 and α7-TRP171(+) [23,43], via cation-π and hydrophobic interactions respectively. In contrast, residues on the complementary interface of rat α7 subunit exert a pivotal impact on the binding affinity of α-conotoxin TxIA (A10L) [51], and residues at the (−) face of human α7 nAChR are also implicated in the determination of selectivity of α-conotoxins to the receptors [23]. Furthermore, in recent studies, Abraham and et al. determined the co-crystal structure of *Lymnaea stagnalis* (Ls) AChBP and LsIA complex [28]; and which was used as the template for building comparative models of LsIA bound to human α7, α3β2 and α3β4 complexes. However, a gap in the knowledge exists regarding the important role of the C-terminal of α-conotoxins in their selectivity and stabilization to nAChRs at the atomic level, and needs further investigation in order to understand and fully exploit the potential of C-terminal modification in designing novel conopeptides for nAChR selective probes and potential drug leads.

In the present study, we elucidate the interaction mechanism of both amidated LsIA and carboxylated C-terminal of LsIA with human α7 using molecular dynamics (MD) simulations together with a number of computational analysis methods. We identified unique intramolecular interactions within LsIA which favored particular conformational motifs for amidated type LsIA, thereby facilitating unique interactions with respective residues of the LBD of α7 nAChR which are absent in carboxylated LsIA. Key pairs of residues have been investigated which are known to be highly implicated in the enhancement of binding potency of amidated LsIA targeting α7 nAChR, compared with its LsIA analogue bound form. We also employed an inter-residue contact network analysis approach for determination of possible disruptions or changes to inter-subunit interactions due to binding of amidated and carboxylated LsIAs, and proposed additional possible structural characteristics which may be related to the higher potency of amidated LsIA relative to carboxylated LsIA.

## 2. Results and Discussion

### 2.1. Homology Modeling of Amidated and Carboxylated LsIAs Anchoring to Human α7 nAChR

The starting homology models of α7/LsIA were constructed using 2BR8 as a template, and subsequently simulated. We note that a recent co-crystal structure is available of *Lymnaea stagnalis* AChBP (*Ls*-AChBP) bound to amidated α-conotoxin LsIA (PDB code: 5T90 [29]), which showed minor structural differences in AChBP compared to all other conotoxin-*Ac*-AChBP crystal structures to date, possibly due to species differences between *Ls*- and *Ac*-AChBP. The authors constructed homology models of amidated LsIA bound to α7 nAChR based on *Ls*-AChBP, and comprehensively elaborated and tested key interactions identified in the model in a number of elegant experiments. Nonetheless, the minor structural differences between *Ls*- and *Ac*-AChBP, and the availability of co-crystal structures of 4/7-conotoxins bound to both species isoforms, afford an opportunity to explore an alternative, possible conformation of LsIA-α7 complex based on *Ac*-AChBP, which shares a comparable (though somewhat lower) sequence similarity to human α7. In this work, we compare and contrast the present LsIA-α7 model based on *Ac*- to that based on *Ls*-AChBP where relevant.

### 2.2. Amidated LsIA Binding Causes Higher Fluctuations in Loop C and the Juxtamembrane Cys-Loop Regions

**Figure 2:**
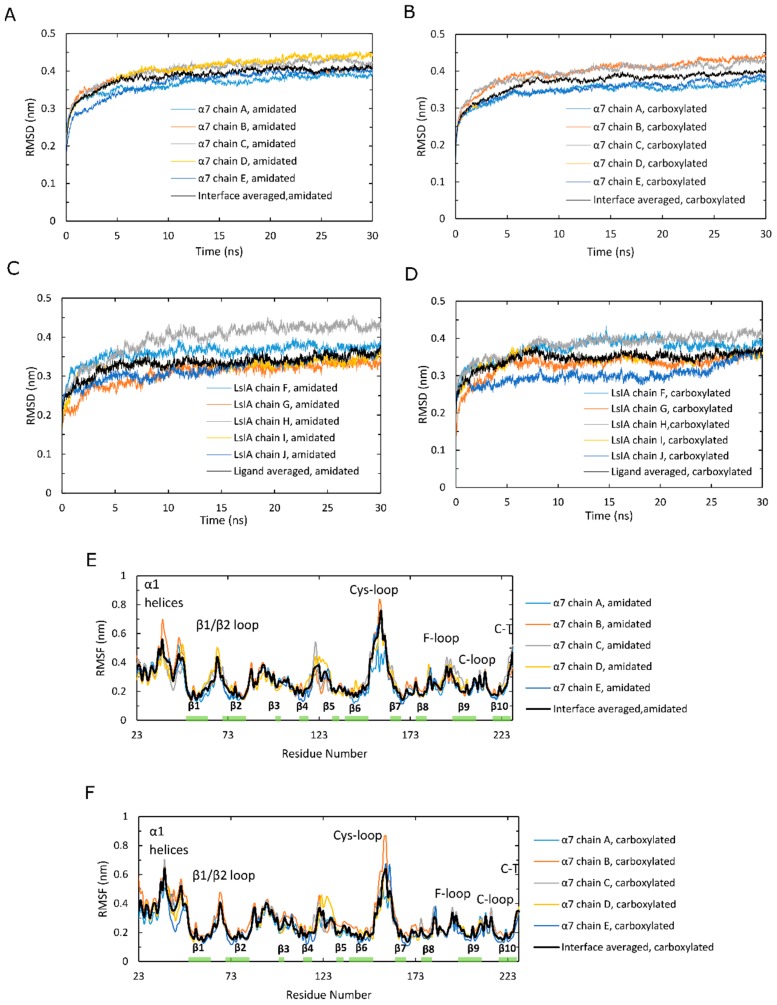

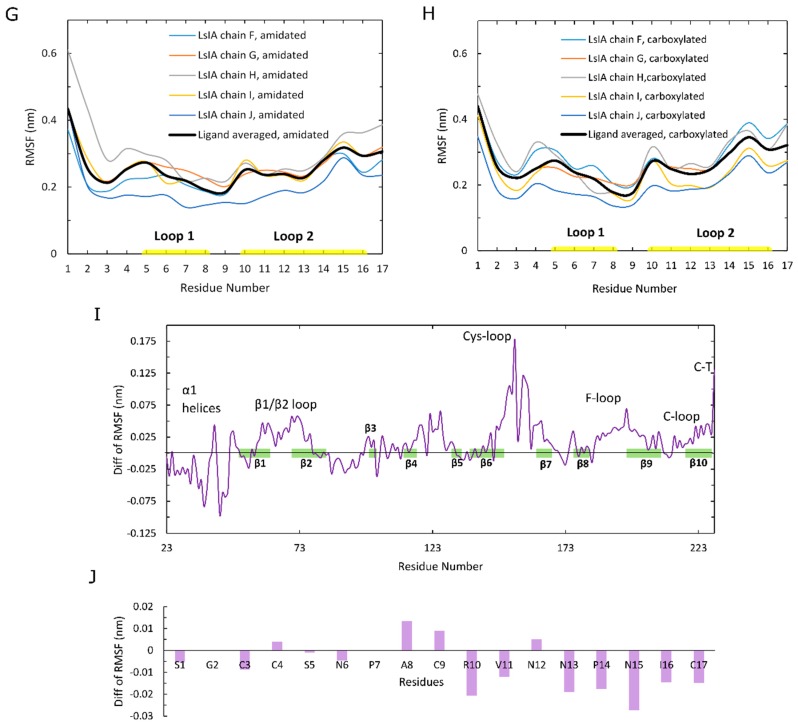
Analysis of stability of human α7 nAChR over molecular dynamics (MD) simulations in the amidated (wt) LsIA and C-terminal carboxylated (mut) LsIA bound types. (**A**,**B**) Cα atoms in β strand RMSD of five α7 subunits in the wt-LsIA and mut-LsIA bound forms, respectively. (**C**,**D**) Cα atoms in β strand RMSD of five ligands (LsIA) binding in the ligand binding domain (LBD) in the wt-LsIA and mut-LsIA, respectively. (**E**,**F**) Cα RMSF of five α7 subunits in the wt-LsIA and mut-LsIA bound forms, respectively. (**G**,**H**) Cα RMSF of five ligands (LsIA) binding in LBD in the wt-LsIA and mut-LsIA, respectively. The black curves demonstrate the mean values of RMSD/RMSF of the five interfaces/ligands in the LsIA binding complexes. (**I**) Differences of Cα RMSF between wt-LsIA and mut-LsIA bound α7 subunits. (**J**) Differences of Cα RMSF between wt-LsIA and mut-LsIA (amidated mean subtracts carboxylated mean).

Time series plots of RMSD, calculated for the backbone atoms and averaged over 12 simulations, for amidated and carboxylated LsIA-α7 nAChR complexes, are shown for the α7 subunits in Figure 2A,B, and for the LsIA toxins in Figure 2C,D. These RMSD plots are qualitatively similar to those of previous simulations of nAChRs-toxins complexes [14,18,52]. The structure of amidated LsIA (Figure 2C) and human α7 nAChR (Figure 2A) complex becomes stabilized, on average, after 20 ns, while the carboxylated LsIA binding at the extracellular domain (ECD) does not achieve its stabilization until 27 ns (Figure 2B,D), and appears to continue to exhibit structural drift towards the end of the simulation period. Thus, there is greater variation in the RMSD plots for the carboxylated LsIA-α7 system compared to amidated LsIA, suggesting that the carboxylated LsIA-α7 complex might be somewhat less stable. This demonstrates a potentially more stable structure of amidated LsIA binding to α7 nAChR complex. This is discussed further in the sections below.

However, the RMSD results show an asymmetric pattern in chain H in amidated LsIA (Figure 2C), which demonstrates a divergent pattern with higher RMSD values (around 0.425 nm) versus the other 4 chains of LsIA, whereas there is no such pattern present in the carboxylated counterpart (Figure 2D). Thus, the amidated LsIA-bound form provides a more steady structure over its analogue binding complex, while the rigidity of protein structure varies in distinct portions of the protein complex (Figure 2C,D). The RMSD plot of chain H (ligand) in amidated LsIA is different from the other chains of the amidated LsIA (Figure 2C). This may suggest a potentially distinct interaction between chain H and corresponding ligand binding domain (LBD) compared with the others. By contrast, we did not observe substantial differences between the five carboxylated LsIA toxins (Figure 2D), nor is there any substantial difference among the chains of α7 nAChR bound by amidated and carboxylated LsIAs (Figure 2A,B). Overall, the RMSD results indicate that amidated LsIA-α7 reach stability earlier, with less fluctuation in RMSD per chain compared to carboxylated LsIA-α7. However, for the amidated complex, there is some asymmetry in the RMSD, with one of the LsIA monomers in the amidated complex undergoing substantially higher structural deviation compared to the others, likely due to LsIA N-terminal (N-T) fluctuations (see below). A similar asymmetric pattern also occurred in the RMSD plot of apo chicken α7 nAChR, determined by Yi and colleagues [15]. RMS fluctuation (RMSF) plots are shown for the amidated and carboxylated LsIA-bound α7 receptor residues in Figure 2E,F, and for the toxin residues in Figure 2G,H, respectively. The absolute RMSF values for the α7 receptors (Figure 2E,F) for both amidated and carboxylated bound forms are consistent with previous studies on human α7. These plots demonstrate the greatest flexibility in the Cys-loop, and are similar to the RMSF results of several previous studies, such as that of α-ImI bound form, by Yu et al. via computational simulations [18], who found that the Cys-loop RMSF increased compared with apo human α7 nAChR. There is also relatively high flexibility of β1/β2 loop and β10 strand, similar to previous studies [53].

In order to clearly show the differences in α7 receptor residue RMSF when bound to amidated compared to carboxylated LsIA, Figure 2I shows “Diff in RMSF”, taken by subtracting the receptor RMSF values of the carboxylated complex from those of the amidated complex. Thus, for example, residues with positive “Diff in RMSF” values indicate higher flexibility for amidated relative to carboxylated LsIA-α7 complex. The main difference is that there is substantially higher flexibility in the Cys-loop region for amidated compared to carboxylated LsIA. This region forms the main contact between the ECD and transmembrane domain (TMD; though absent in the present simulation) in the full α7 nAChR. Enhanced flexibility in the Cys-loop regions could be related to changes in α7 channel activity, and could provide an indirect measure of the potential inhibitory effects of conotoxins bound to nAChR ECDs. Further work involving the whole α7 receptor bound to conotoxins will likely be required to examine the direct impact of toxin binding on the pore region. Another region that exhibits marginally higher RMSF for amidated LsIA-bound α7 is the loop C region, which forms part of the canonical agonist binding site and, in the α-subunits of nAChRs, is composed of a β hairpin with a twin Cys motif at the edge of the loop. This enhanced fluctuation is in accordance with previous studies on nAChRs, including those for apo α7 and α7-antagonist-bound complexes [53]. Nonetheless, the higher RMSF of both loop C and the juxtamembrane Cys-loop region, for amidated LsIA, is consistent with the present notion that movements in these two areas are strongly coupled, and is required for transmission of structural effects due to agonist (or antagonist) binding from the ECD to the transmembrane pore. The higher RMSF for both these regions for amidated LsIA suggests that this natural form of the conotoxin may have a greater impact on the ligand binding and subsequent TMD coupling mechanism of α7 compared to the less potent, carboxylated form.

There are also slightly higher RMSF values in regions of the β1/β2 loop and C-terminus of β10 strand that are related to pore gating in the TMD. Both regions are related to the contacts between ECD and TMD for mediating the open and closed states of the pore in TMD, but via distinct pathways. The increased flexibility of the former regions that interact with M2–M3 linker may cause a conformational change of M2 α-helices in the TMD [54,55,56], which is a known role of the Cys-loop in pore gating of Cys-loop receptors [57,58]. The latter region is associated with the N-terminus of M1 in the TMD via covalent interactions [59].

Additionally, there is reduced flexibility in the N-terminal α1 helices of nicotinic α7 nAChR bound by amidated LsIA relative to carboxylated LsIA. This region has been demonstrated to form a key Fab35 antibody binding region for muscle-type nAChR subtype [60], but its role in neuronal nAChRs is presently less clear. The impact of conotoxin binding on nAChR-antibody linkage is an intriguing future avenue of research. The absolute RMSF plots for the toxin residues of amidated and carboxylated LsIAs are shown in Figure 2G,H respectively. Both exhibit similar patterns of RMSF, with especially high fluctuation at N- and C-terminal residues. As with the RMSD behavior described above, for amidated LsIA, there is some asymmetry in RMSF, with one monomer (labeled chain H in Figure 2G) exhibiting substantially higher fluctuation in the N-terminus compared to all four of the other monomers. Thus, the source of asymmetry appears to be localized mainly to the disordered and highly flexible loop 1 residues, SER1 and GLY2.

To directly compare the effects of carboxylation, the “Diff in RMSF” plot for the LsIA residues is shown in Figure 2J. It can be seen that residues ALA8 and CYS9 are more flexible for the amidated LsIA, with positive “Diff in RMSF” values. However, the main impact is that amidated LsIA has a more rigid structure in residues of loop 2 (except for ASN12) and in the N-terminus of LsIA (Figure 2G,H,J). The rigidity in loop 2 is due to differences in both toxin-receptor complexes, as well as intramolecular LsIA contacts, as detailed below.

### 2.3. Carboxylation at LsIA C-Terminus Causes Indirect Loss of Contacts between Key Residue ARG10 and the α7 Complementary Face

**Figure 3:**
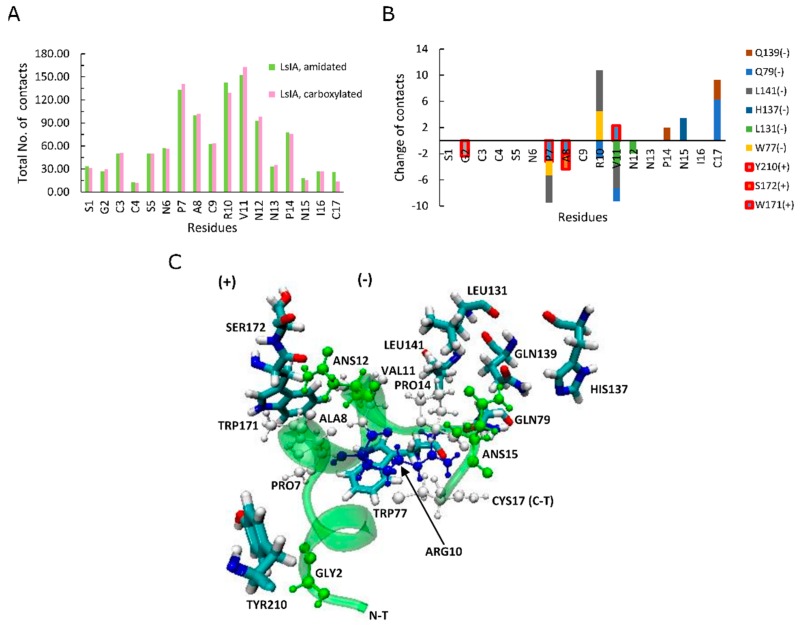
A total number of contacts and the variations between amidated (wt) LsIA and C-terminal carboxylated (mut) LsIA bound types. (**A**) The total number of contacts for amidated (*green*) and carboxylated (*pink*) LsIA with human α7 nAChR. (**B**) The significant changes (Amid minus Carb) in the number of contacts between wt-LsIA and mut-LsIA bound types. (**C**) The interactive residues of the wt-LsIA and the α7 nicotinic receptor complex.

To explain differences in RMSD and RMSF, we examined how amidation and carboxylation change the extent of residue pairwise contacts between LsIA and α7. Figure 3A shows the absolute total number of contacts between each residue of amidated (green) and carboxylated (pink) with α7, as well as differences (amidated minus carboxylated, Figure 3B). The most significant interactions formed by amidated and carboxylated LsIAs with α7 subunit, which predominantly occurred in ARG10 and VAL11 in loop 2 and LsIA-PRO7 in loop 1, which is also known to exert a great impact on selective targeting by the ligand (Figure 3A) [28,61]. Other minor interactions are established by LsIA-CYS17 and the residues on the receptor, significantly contributing to the contacts of LsIA (Figure 3A,B). In contrast, although LsIA-ALA8 makes substantial contacts with the receptor, its impact on selectively binding to the target is much lower when compared with other residues of LsIA, as explained below.

Figure 3B shows differences in the number of contacts for the LsIA residues. The effects of gaining contacts for amidated, relative to carboxylated LsIA, outweighed those of losing contacts on the LBD of human α7 nAChR subtype in total, as also suggested by Inserra et al. in their experimental results [29]. However, there were no significant changes appearing in loop 1 of LsIA, while most of the variations of contacts took place in loop 2 and C-terminus of LsIA (Figure 3B). Since loop 2 makes contacts mostly with α7(−) (Figure 3B,C), this pattern indicates that the CT amidation/carboxylation state mainly affects interactions with the α7(−) face. Enhancement in toxin-receptor contacts for the amidated form is qualitatively consistent with this toxin’s higher potency compared to the carboxylated form. Two residues in loop 2 show substantially higher contacts for the amidated form; ARG10 and CYS17 (Figure 3B). This is consistent with the predicted importance of ARG10 in forming close hydrogen bonds with the hydrophilic pocket of α7 in previous recent studies [28]. Compared with the carboxylated LsIA bound form, although wt-LsIA-ARG10 shows a higher absolute number of contacts with α7 nAChR, in relative (percentage) terms, wt-LsIA-CYS17 has the greatest enhancement, with an increase of 45% higher contact number compared with the other residues of amidated LsIA (not shown). On the contrary, the most significantly impaired interaction was attributed to GLY2 with around 20% decrease (not shown). Additionally, PRO7, VAL11 and ASN12 show minor reductions for amidated LsIA and α7 nAChR complex, but the contacts of ASN in position 15 and PRO14 with the receptor are slightly enhanced in the amidated ligand-bound form. Overall, then, for the amidated LsIA, an increase in the number of contacts at ARG10 and the C-terminus with α7 (Figure 3B) is likely responsible for its higher rigidity in loop 2 (Figure 2J, RMSF plots) compared to the carboxylated form. Since ARG10 is known to be a key residue responsible for LsIA inhibition of nAChRs [28], our present simulation results indicate that introducing a negative charge via carboxylation at the C-terminus not only causes reduction in contacts directly with CYS17, but also an indirect loss of contact at ARG10, and this latter disruption may play the key role in lower potency of the carboxylated LsIA at α7.

### 2.4. Amidated LsIA Favors Hydrogen Bonding and π-π Interactions between ARG10 and the α7(−) Subunit

We examined specific toxin-receptor pairwise contacts, which may play an important role in improving the selectivity of amidated LsIA binding to human α7 nAChR, namely LsIA-CYS17 with α7-GLN79(−) and GLN139(−), and LsIA-ARG10 with α7-LEU141(−) and TRP-77(−) (Figure 3C). In contrast, PRO7 and VAL11 of carboxylated LsIA possess preferential contacts with residues on the principal (+) face of α7. We first discuss interactions which favor the amidated LsIA, followed by those that favor carboxylated LsIA.

**Figure 4:**
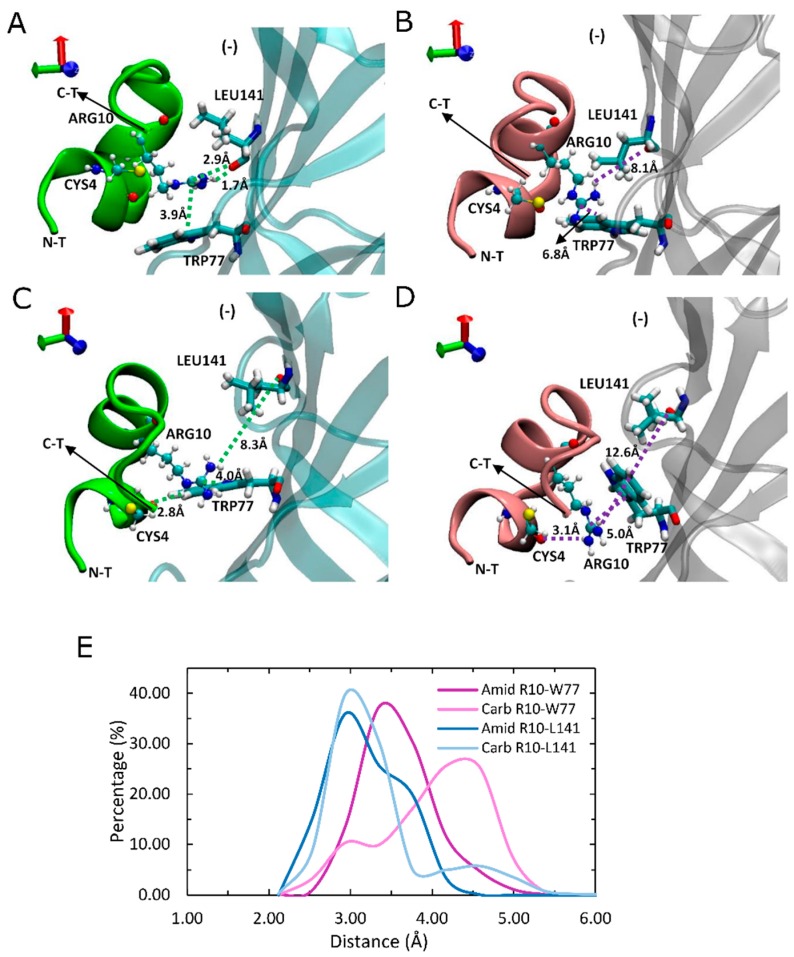
Side view of binding conformation of LsIA-ARG10 to the ligand binding domain (LDB) of human α7 nAChR. (**A**) The sandwich-like motif of amidated (wt) LsIA-ARG10 with α7-LEU141(−) and TRP77(−) and (**B**) binding conformation of carboxylated (mut) LsIA with corresponding residues. (**C**,**D**) Perpendicular-like binding conformation of LsIA-ARG10 with α7 LEU141 and TRP77 in amidated (**C**) and carboxylated (**D**) LsIA bound form, respectively. The *purple* and *green* dash lines indicate the residues of LsIA with impaired and improved interaction with respective residues of the receptor, respectively, whereas the receptor subunits bound by wt-LsIA (*green*) and mut-LsIA (*pink*) are shown in transparent *cyan* and *silver* colors, respectively. (**E**) Distances (Å) of the corresponding interactions between residues of LsIA and receptors in ligand-bound forms via gmx_mindist.

For amidated LsIA, a significant interaction is established between LsIA-ARG10 and α7-TRP77(−) and LEU141(−) via hydrogen bonds and cation-π interaction, respectively, via a sandwich-like arrangement (Figure 4A), which was observed in the amidated but not carboxylated (Figure 4A,B), LsIA. Compared with the carboxylated analogue and α7 nAChR complex, a unique sandwich-like conformation [62], composed by wt-LsIA-ARG10 and α7-TRP77(−) (3.9 Å) and α7-LEU 141(−) (1.7 Å/2.9 Å) (Figure 4A), was determined, and this group of interactions might serve to stabilize interaction of ARG10 with residues at α7(−), leading to a deeper burial of amidated LsIA into the binding pocket. A similar ‘sandwich’ motif has also been determined by Grishin and colleagues through computational methods [63], demonstrating a particular arrangement for AulB-F9 and aromatic residues, LYS61 and TRP59, in the β4 subunit of α3β4 nAChR. However, the interactions involved in the present study also include hydrogen bond formation.

We also observed a perpendicular (T-shaped) position of the LsIA-ARG10 sidechain with respect to α7-TRP77(−) in both LsIA bound conformations (Figure 4C,D), wherein both cation-π interactions are likely to be relatively weak in this arrangement (4.0 Å in amidated LsIA; 5.0 Å in carboxylated LsIA). The intramolecular interactions between CYS4 and ARG10 may account for this situation, as this T-shaped motif often occurred when LsIA-CYS4 lost its persistent contacts with the guanidinium moiety of ARG10. Nevertheless, this perpendicular conformation is relatively rare in the wt-LsIA bound form, due to the tight intramolecular interactions.

Furthermore, the cation-π interaction has also been demonstrated between ARG7 of α-ImI and aromatic residues, such as TYR217(+), TRP171(+) and TYR115(+), on the binding pocket of human α7 nAChR through computational docking results [64]. In addition, as TRP77(−) significantly affected the specific binding of α-ImI to human α7 nAChR and was shown to be crucial for modulating the response of rat α7 nAChR to the existence of agonist [43,65], further investigation on binding of LsIA at human α7 nAChR may help to explicate the important role of TRP77(−) in LsIA binding. The preferential interactions involving hydrogen bonding between ARG10 and the hydrophilic environment of the α7(−) subunit is in qualitative agreement with the model proposed by Abraham et al. [28].

**Figure 5:**
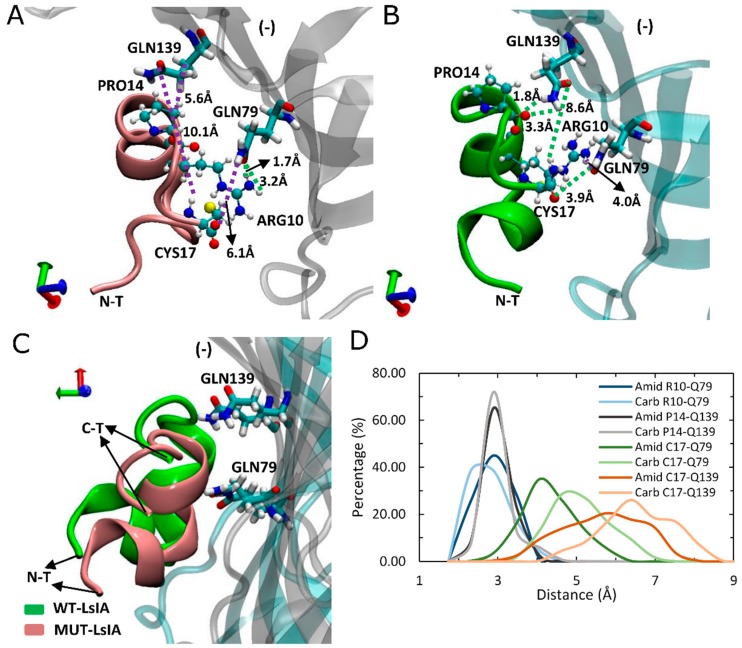
Binding model of LsIA-PRO14, ARG10 and CYS17 of amidated (wt) LsIA and C-terminal carboxylation (mut) LsIA into the binding pocket on the complementary (−) face of human α7 nAChR. (**A**,**B**) Interactions established by ARG10, PRO14 and CYS17 with corresponding residues in amidated (*green*) and carboxylated (*pink*) LsIA binding forms. (**C**) Side view of the binding conformation of the wt-LsIA and mut-LsIA to α7 nAChR subunit. (**D**) Distances (Å) of the corresponding interactions between residues of LsIA and receptor in ligand-bound forms.

Other, more minor interactions identified in the present simulations include those between the amidated-LsIA-CYS17 with GLN139(−) and GLN79(−) (Figure 5A,B) as well as HIS137(−) (not shown), of α7 subunit, via hydrogen bond interactions. Notably, interactions between CYS17 and the respective GLN residues of the receptor, are amongst the most significantly enhanced contacts, compared with its carboxylated counterpart bound complex (Figure 3B). However, it should be noted that although the distance between amidated LsIA-CYS17 and GLN139(−) is decreased, the interaction between them is likely to be relatively weak, as a hydrogen bond interaction is not possible due to a long distance (8.6 Å) between LsIA-CYS17 and GLN139(−). The proximate packing of wt-LsIA (Figure 5C) allowed a minor persistent hydrogen bond interaction between contacts of PRO14 and GLN139(−) (1.8 Å/3.3 Å) (Figure 5B,D), whereas a relatively weak van der Waals interaction is established with corresponding residues in the carboxylated LsIA binding form (Figure 5A).

### 2.5. Carboxylated LsIA Favors Hydrophobic Interactions with α7 Subunit Residues via PRO7 and VAL11

The description in the above section noted the loss of amidated LsIA contacts between PRO7 and ALA8 with α7-TRP171(+), and VAL11 with α7-LEU131/141(−) (Figure 3B) which are, conversely, favored by the carboxylated LsIA. This may be related to the shifting of the balance in LsIA interactions with the principal (+) and complementary (−) subunits mentioned above.

**Figure 6:**
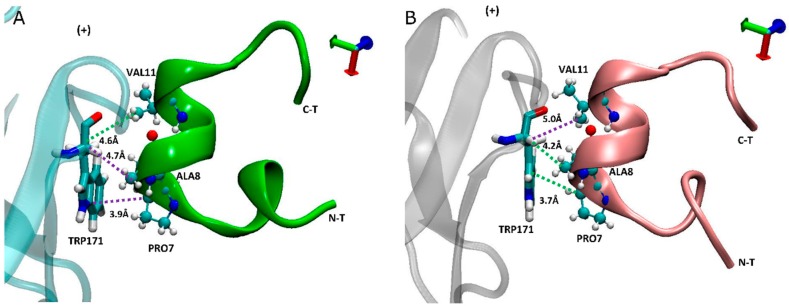
Side view of binding conformation of LsIA into the binding pocket with the interaction between human α7-TRP171(+) and LsIA-PRO7, ALA8 and VAL11, respectively. (**A**) The conformation of amidated LsIA anchored to the principal (+) face of α7 subunit. (**B**) The conformation of carboxylated LsIA anchored to the (+) face of α7 subunit.

In addition to α7-TRP171(+) having higher contacts with carboxylated LsIA-PRO7 and LsIA-ALA8 (Figure 6), we noted that the interaction between TRP171(+) and LsIA-PRO7 also shows the highest absolute number of contacts in both amidated and carboxylated LsIA bound receptors (not shown). Proline in position 7 of LsIA, which is amongst the conserved amino acids of LsIA, played a critical role in the formation of the α-helix structure of α-conotoxins, by strongly contributing to binding of α-conotoxins at nAChR receptor through making various contacts with residues on both sides of the binding site [1,43,64]. From the inter-residue contact results, the highest number of contacts are formed between α7-TRP171(+) and carboxylated LsIA-PRO7 due to hydrophobic interaction between TRP171(+) and the imino ring of carboxylated LsIA-PRO7, the interaction of which has also been identified between α7-TRP171(+) and PnIB-PRO6 and PRO7 in previous experimental studies of other conotoxins [4,23]. Furthermore, VAL11 forms hydrophobic contacts with the residues LEU131/141(−), further contributing to enhanced hydrophobic interactions relative to amidated LsIA.

### 2.6. α7-TYR217 Forms Close Contacts with Both Amidated and Carboxylated LsIAs

**Figure 7:**
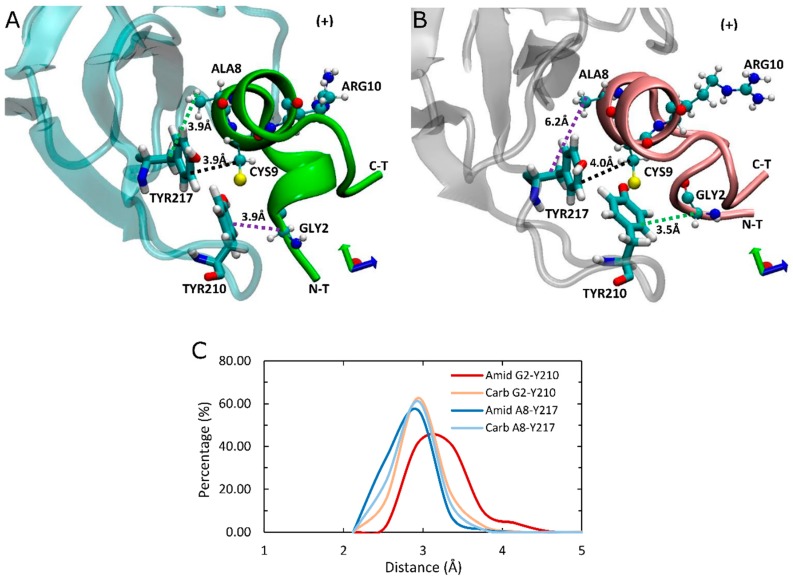
Top view of binding conformation of amidated (wt) and carboxylated (mut) LsIA at the principal (+) face of human α7 nAchR. (**A**) The conformation of wt-LsIA anchored to the (+) face of α7 subunit. (**B**) The conformation of mut-LsIA anchored to the (+) face of α7 subunit. The black dash lines demonstrate the interaction occurred in both LsIA bound forms. (**C**) Distances (Å) of the interactive residues in LsIA and the LBD of α7 nAChR complexes.

As the principal determinant for binding of ImI is the interaction between aromatic ring of α7-TYR217(+) of receptor and ARG6 [43], we also investigated the interaction between α7-TY217(+) and respective residues of LsIA. However, there is no potential interaction between LsIA-ARG10 of amidated or carboxylated LsIA and the corresponding residue; nor are apparent variations of interactions between these two residues detected, due to the position of LsIA-ARG10 being relatively far away from α7-TYR217(+) (Figure 7A,B). Nonetheless, several contacts were identified in the present simulations, between α7-TYR217(+) and LsIA-ALA8, LsIA-CYS9 and the two ASN residues in loop 2 of the toxin, which were observed in both amidated and carboxylated LsIAs. Among them, the most notable interaction is established by LsIA-CYS9 with α7-TYR217(+). CYS9 is in close proximity with TYR217(+), which allows van der Waals interaction that could be an important determinant for binding LsIA at α7 nAChR (Figure 7A,B). Additionally, hydrophobic interaction formed by α7-TYR217(+) and LsIA-ALA8 also may be related to the anchoring of LsIA and its variant to human α7. However, there are no obvious changes in contacts between TYR217(+) and CYS9 regarding carboxylating the C-terminal of LsIA, whereas the degree of hydrophobic interaction between LsIA-ALA8 and TYR217(+) (3.9 Å) is slightly higher in amidated LsIA receptor complex compared with the carboxylated LsIA (6.2 Å) (Figure 7). In contrast, the van der Waals interaction established between LsIA-GLY2 and α7-TYR210(+) is reduced, possibly owing to the amidated LsIA being more deeply inserted into ligand binding pocket, together with a more flexible backbone of the N-terminal of amidated LsIA, relative to its carboxylated analogue bound form.

### 2.7. An Intramolecular Salt-Bridge in Carboxylated LsIA Is Retained Upon Binding to α7, and Plays a Role in the Loss of Key Toxin-Receptor Contacts

**Figure 8:**
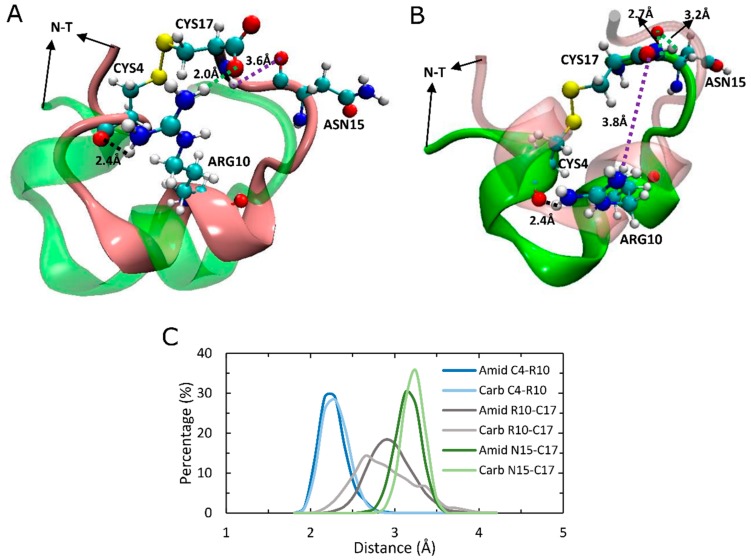
The intramolecular interactions within C-terminal carboxylated (mut)and amidated (wt) LsIAs by binding to human α7 nAChR. (**A**) The conformation of mut-LsIA (**B**) The conformation of wt-LsIA. The conformation of wt-LsIA in (**A**) and mut-LsIA in (**B**) is shown in transparent *green* and *pink* colors, respectively. (**C**) Distances (Å) of intramolecular interactions between residues of wt-LsIA and mut-LsIA.

While C-terminal amidation is known to be important for the folding of α-conotoxin ImI through its effects on disulfide pairing [66], it may also directly or indirectly affect the binding stability of LsIA to human α7 nAChR via intramolecular interactions. The present simulations indicate that the carboxylated LsIA-CYS17 forms a stable salt bridge (2.0 Å) with ARG10 (Figure 8A), the interaction of which was also discovered by Inserra and colleagues in experiments [29] in the solution NMR structure of the free solvated LsIA. Thus, our present simulation confirms that this CT-ARG10 salt bridge remains intact when LsIA is bound to α7 also.

In addition to this salt bridge interaction, our simulations identified a number of additional persistent hydrogen bonds which may further differentiate amidated from carboxylated LsIA. Another persistent hydrogen bond formed by amidated LsIA-CYS17 and the backbone carbonyl group of ASN15 in both types of LsIA bound to α7 was also observed in the present simulations (Figure 8). This intramolecular interaction is likely involved in drawing the C-terminus closer to loop 2 of α-helix of LsIAs (Figure 8A,B). Furthermore, the amidated LsIA-ASN15 also forms hydrogen bonds with the backbone of CYS17, with distances of 2.7 Å and 3.2 Å (Figure 8B). This additional hydrogen bonding pattern may play a role in the greater rigidity of CYS17 and ASN15 shown in the RMSF plots for amidated LsIA over its carboxylated analogue (Figure 2J). Finally, a persistent intramolecular hydrogen bond is also formed by ARG10 with the backbone of CYS4 (2.4 Å) in both amidated and carboxylated LsIAs, and may play a role in maintaining LsIA in its characteristic fold for both analogues.

### 2.8. Changes in Inter-Subunit Contacts on the Five Interfaces of Adjacent α7 Subunits

**Figure 9:**
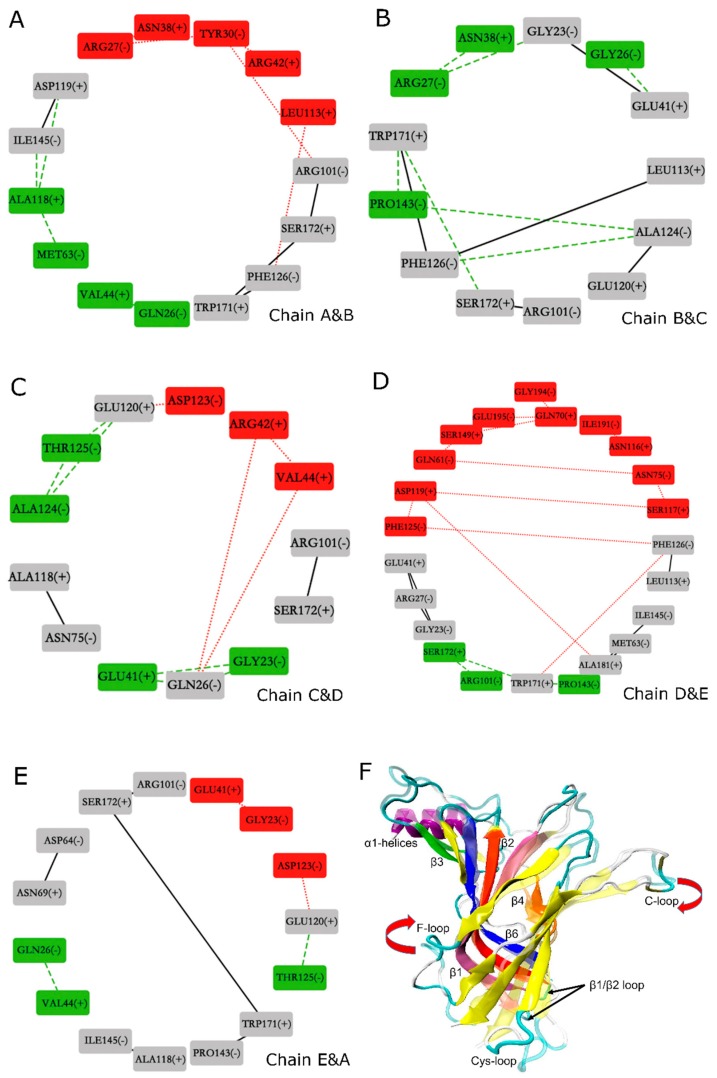
Comparison of differences in the interactions between principal (+) and complementary (−) interfaces of human α7 nAChR after binding of amidated and carboxylated LsIAs. (**A**–**E**) Determination of interactive residues at five interfaces of α7 nAChR via Cytoscape program. (**F**) Comformation of changes in the secondary structure of (+) face of LsIA bound receptors, where amidated LsIA bound face is shown in transparent color.

The binding of amidated and carboxylated LsIAs to human α7 also elicits variations of contacts between residues at the interfaces between subunits of α7, which were examined using the Cytoscape [67] molecular analysis method. It is hereby proposed that structural disruptions to the integrity of inter-subunit contacts may have implications for the potency of conotoxins against nAChRs. Figure 9 shows the network representations of changes in inter-subunit contacts for the nodes (boxes) indicate interface residues, numbered and labeled by receptor chain (A–E). Each sub-figure shows residues at a different interface (e.g., Figure 9A shows the chain A–B interface, while Figure 9B shows the chain B–C interface). The edges (lines) connecting the nodes indicate possible contacts between the interface residues; red dot lines indicate contacts that are closer for the carboxylated form, green dash lines indicate contacts that are closer for the amidated form, while black lines indicate contacts that are similar for both forms. The nodes are color coded as follows: residues are colored red if they mainly have closer contacts with opposing interface residues at the *carboxylated* form, green if they mainly have closer contacts with opposing interface residues at the *amidated* form, and gray if their overall contacts with opposing residues are unchanged between the two forms.

Overall, the predominance of grey nodes and edges shows that amidation generally caused similar inter-subunit contacts to C-T carboxylation. There is a slightly closer association between the interfaces for the amidated form, since all interfaces contain some residues that have closer association at the amidated form indicated by the presence of green nodes in all Figure 9A–E; but the interface between chains B and C does not have any residues that associate more closely at the carboxylated form, indicated by the absence of red nodes in Figure 9B. Nonetheless, certain interactions within interfaces of the carboxylated bound form which are weakened may have implications for LsIA binding to the LBD. On the other hand, interface contacts weakened by binding of amidated LsIA may also have implications for the inhibitory potency of this form of the toxin. Below, more details regarding which residues were involved in these disruptions in inter-subunit contacts, are discussed.

In general, acidic residues (ASP and GLU) at the inter-subunit interfaces have higher contacts with other residues for the amidated form. This can be demonstrated by considering certain residues situated in the N-terminal α1 helix(−) which interacted with the residues on loop between α-helix and β1(+), and resulted in pulling the N-terminal helices into a closer proximity in both amidated and carboxylated LsIA forms, and the gap between interfaces, therefore, was slightly shortened by toxin binding. For example, GLN26(−), were dominantly involved in interacting with both VAL44(+) (chains A&B and chains E&A) (Figure 9A,E) and with GLU41(+) (chains B&C and chains C&D) (Figure 9B,C), respectively. Interestingly, repulsive electrostatic interaction established by GLU120(+) and ASP123(−) (chains C&D and chains E&A) in carboxylated bound type, may elicit separation of the conjunctive interfaces (Figure 9C,E). In this case, residues on carboxylated LsIA probably lost contacts with the receptor due to the close proximity of several negative charges contributed by LsIA as well as the acidic residues of the two interfaces of the receptor. Additionally, on the boundary surface between chain D(+) and E(−) (Figure 9D), GLY194(−), GLU-195(−) and ILE-191(−) forms more interactions with GLN70(+) and ASN116(+) on β2 and β4 strands, respectively, in carboxylated bound form. These observed interactions may be related to a motion which may be described as an upward swing of the F-loop and a downward shift of the C-loop of chain E (Figure 9F), which has been suggested to promote agonist binding at apo chicken α7-nAChR [15]. Furthermore, β6 and C-loop have been determined as part of key determinants for binding of ACh to α7 nAChR [18].

Finally, another trend exhibited by examination of the network diagrams shows that several clusters of hydrophobic interactions form closer contacts with each other in the amidated form. Specifically, PRO143(−) on β6(−), forms hydrophobic interaction with TRP-171(+) (chains B&C and chains D&E) respectively on B-loop(+), shown in Figure 9B,D, which results in a longer distance between TRP-171(+) and S172(+) with PRO7 and ALA8 in amidated LsIA by triggering the shifting of B-loop(+) toward β6(−). Another unique hydrophobic interaction was determined between ALA118(+) and MET63(−) and ILE145(−) respectively, on chain A and B interface (Figure 9A). Among them, methionine residue is situated close to β1 and β2 loop, that were implicated in the conformational changes of the TMD of AChBP [68].

Taken together, the conclusions that can be drawn are that (1) binding of amidated LsIA favors inter-subunit interactions involving ASP and GLU near the agonist-binding site; and (2) binding of carboxylated LsIA favors a closer association between hydrophobic residues centered on PRO143(−) and ALA118(+), near the Cys-loop regions at the juxtamembrane domain. However, further studies are required for exploring the effects of the inter-α7 subunit contacts on stabilization and specificity of LsIA binding to the receptor.

## 3. Conclusions

In order to understand the importance of C-terminal of LsIA for selectively binding to human α7 nAChR in atomic level, we employed MD simulations to propose the determinants for the differential activity between naturally amidated and carboxylated LsIAs at the α7 nicotinic receptor. Simulations suggest that amidated LsIA-bound α7 exhibits higher fluctuations in loop C (at the canonical agonist binding site), as well as the juxtamembrane Cys-loop region. This variation in dynamic fluctuations in both regions together may be related to differences in the capability of structural changes to be transmitted from the ECD down to the transmembrane domain when amidated, but not carboxylated, LsIA is bound to α7. Our findings also confirm a persistent intra-molecular salt bridge between ARG10 and the C-terminus in carboxylated LsIA is retained when bound to α7, and may be responsible for preventing the interactions between ARG10 and key residues in α7, such as ARG10 and TRP77(−) and LEU141(−), via cation-π and hydrogen bond interactions in amidated LsIA bound form, and therefore, affects the preferential selectivity of amidated LsIA to α7 nAChRs,. This model is qualitatively consistent with the previous experimental findings which proposed a key role for ARG10 in forming interactions within the hydrophilic environment of the α7 binding pocket. Overall, carboxylation of the C-terminal reduces the selectivity of LsIA to α7 nAChR versus its native type.

Additionally, the impacts of binding of amidated and carboxylated LsIAs on the overall structure and inter-subunit contacts were examined using an inter-residue network analysis approach, suggesting a clockwise tilting of C and F loops upon binding to carboxylated LsIA, which is absent for amidated LsIA binding. The network analysis also indicates that proximate acidic residues ASP and GLU are possible at interfaces of α7 nAChR bound by the amidated LsIA, whereas the carboxylated counterpart disrupts these same-charge contacts while, instead, promoting closer contacts between interfacial hydrophobic residues. This approach may facilitate the determination of structural variations or changes in residue interactions at inter-subunit interfaces of the receptor upon binding of peptides.

Further exploration may be required for understanding specific impacts of residues of LsIA-α7 nAChR complexes on selectivity and stability of the ligands and the influence of inter-subunit contacts on native LsIA, via free binding energy calculation and experimental mutagenesis studies to probe the effects of the key residues, at both LsIA and α7, which are proposed in this present work. Furthermore, the carboxylation of C-terminals of conotoxins with known activity at receptors may need investigation in the future, such as studies on their binding potency and interactions with nAChRs or structural stability. The predicted molecular mechanism of LsIA binding to the neuronal nAChR, and in particular the possible roles of the C-terminal charge state, may facilitate a better understanding of the effect of C-terminal amidation/carboxylation on the binding potency of conotoxins at neuronal nAChRs, as most α-conotoxins and certain neuroactive peptides in other conotoxin subfamilies also possess a naturally amidated C-terminal in their structures. More broadly, these findings may assist in the design of novel peptides for nAChRs related disease and nAChR selective probes with high potency for understanding the structural and pharmacological features of nAChRs and other ion channels.

## 4. Methods

### 4.1. Homology Modeling

The sequence alignment of human α7 nAChR (UniProtKB:P36544) to *Ac*-AChBP was determined using multiple sequence alignment of human nAChR subunits α4-10 and β2-4 with that of *Aplysia californica* acetylcholine binding protein (*Ac*-AChBP). Subsequently, homology models of amidated LsIA bound to the pentameric extracellular domain (ECD) of human α7 were generated using Modeler9v6 (Sali and Blundell, 1993) using the coordinates of *Ac*-AChBP co-crystallized with the double mutant α-conotoxin PnIA[A10L,D14K] (Protein Data Bank (PDB) accession code 2BR8) [6] as a template. One hundred models were generated, after which the model with the top DOPE [69] score was selected for simulations. The modification of the C-terminus of LsIA was conducted using Deep View (Swiss-PdbViewer, v4.1, Swiss Institute of Bioinformatics, Lausanne, Switzerland) [70] for changing the naturally amidated C-terminal to the carboxylated one.

### 4.2. Molecular Dynamics Simulations

The homology models of amidated and C-terminal carboxylated LsIAs anchored to human α7 nAChR, were centered in rectangular boxes with an initial size of 99.593 × 99.593 × 99.593 Å^3^ and 99.603 × 99.603 × 99.603 Å^3^, respectively, for fully submerging the α7-LsIA complex structures in water. Both of the systems were energy minimised using Gromacs 4 (v4.6.5, Science For Life Laboratory, Stockholm University and KTH, Stockholm, Sweden; and Biomedical Centre, Uppsala, Sweden) [71] with the CHARMM27 forcefield and were solvated with water by TIP3P water model [72]. Na^+^ and Cl^−^ ions were added for achieving a concentration of 0.15 M and neutralizing the charged amino acid residues in the two systems. For LsIA-α7 complex, 80 Na^+^ and 70 Cl^−^ were added, whereas 85 Na^+^ and 70 Cl^−^ were introduced in the carboxylated LsIA bound complex. Both systems were energy minimized for up to 1000 time steps utilizing the steepest descent minimisation algorithm. The temperature was regulated by the velocity-rescale method [73,74], increasing from 0 K to 300 K over 100 ps in NPT ensembles with pressure under 1 atm. In order to restrain all protein molecules in the systems, the LINCS algorithm [75] was implemented with a time step of 2 fs. The cutoff of van der Waals interaction was set to 12 Å with a smooth switch of 10 Å, meanwhile, the electrostatic energy was calculated via Particle Mesh Ewald (PME) method [76]. Subsequently, the production runs under constant NPT conditions were conducted for at least 30 ns with restraints removed. Production simulations of both amidated and carboxylated LsIA-α7 complexes were performed using different random seeds to set random initial particle velocities, with 12 independent simulations for each system. Since α7 nAChR contains five equivalent interfaces, the total amount of trajectory collected for analysis of α7(+)α7(−) interfaces were at least 3.6 microseconds (2 systems × 12 independent simulations × 30 ns × 5 interfaces). Analyses were performed either on data obtained as an average over the independent simulation trajectories, or over independent trajectories as well as interfaces, as noted in the Results and Discussion. In this study, the frames were saved every 10 ps for analyses for all trajectories.

### 4.3. Structural Visualization and Analysis

Conformations of the LsIA-α7 complexes obtained by MD simulations, were identified and analysed using a number of computational methods. Visual Molecular Dynamics (VMD) [77] was used for observing MD simulation results and production of figures of binding conformations. Root-mean-square deviation (RMSD) and RMS fluctuation (RMSF) were calculated using the GROMACS suite of analysis tools; and were used for evaluation of structure stability, flexibility and rigidity of the protein complexes. The average structures generated from the *rmsf* command implemented in GROMACS were examined using Cytoscape [67], which is a program for visualization and comparison of inter-residue interaction networks in protein complexes. The *mindist* command implemented in GROMACS was used for determining numbers and distances of contacts between interactive residues of ligand and receptor with a cutoff of 4.5 Å. In all Results and Discussion sections, the five subunits of α7 nAChR are labeled chains A to E, while the five amidated or carboxylated LsIA conotoxins binding at the interfaces between the α7(+)α7(−) subunits are labeled chains F to J.

RMSD, RMSF and gmx_mindist analysis are based upon different manipulations of MD simulation trajectories. RMSD of each subunit/ligand was the average based on 12 seeds of MD simulation trajectories with each seed running for around 40 ns with the segment beyond 30 ns cut off. Therefore, the total amount of time shown in Figure 2A–D is 30 ns. The RMSF of each subunit/ligand was calculated based upon the original MD simulation data. 7a and 7c are short for amidated LsIA and carboxylated LsIA bound form, respectively. The Gmx_mindist of each paired residue was calculated based upon the whole trajectory, with the first 10 ns of each individual seed being cut off.
